# Relationship between 24 h blood pressure variability and mortality in acute myocardial infarction patients

**DOI:** 10.1002/clc.24261

**Published:** 2024-04-02

**Authors:** Ying Liu, Wen Li, Shuoyan An, Zhengqin Zhai, Xinju Liu, Mengxue Hei, Gailing Chen

**Affiliations:** ^1^ Department of Cardiology China‐Japan Friendship Hospital Beijing China; ^2^ China‐Japan Friendship Hospital Clinical Medical College Beijing University of Chinese Medicine Beijing China; ^3^ The Second Clinical Medical College Xinjiang Medical University Urumqi China

**Keywords:** blood pressure, mortality, myocardial infarction

## Abstract

**Background:**

In recent years, the mortality of patients with AMI has not declined significantly. The relationship between blood pressure variability (BPV) and acute myocardial infarction (AMI) is unclear. We explored the relationship between 24‐h BPV and mortality in patients with AMI.

**Hypothesis:**

The mortality of patients with AMI is related to BPV. We hope to provide therapeutic ideas for reducing the risk of death in patients with AMI.

**Methods:**

This is a retrospective cohort study. We extracted and analyzed data from the MIMIC‐IV 2.0, which was established in 1999 under the auspices of the National Institutes of Health (America). The average real variability (ARV) was calculated for the first 24‐h blood pressure measurement after patients with AMI were admitted to the intensive care unit (ICU). Patients were divided into four groups according to ARV quartiles. The outcomes were 30‐day, 1‐year, and 3‐year all‐cause mortalities. Data were analyzed using Cox regression, Kaplan–Meier curves, and restricted cubic spline (RCS) curves.

**Results:**

We enrolled 1291 patients with AMI, including 475 female. The patients were divided into four groups according to the qualities of diastolic blood pressure (DBP)‐ARV. There were significant differences in the 30‐day, 1‐year and 3‐year mortality among the four groups (*p* = .02, *p* < .001, *p* < .001, respectively). After adjustment for confounding factors, systolic blood pressure (SBP)‐ARV could not predict AMI patient mortality (*p* > .05), while the highest DBP‐ARV was associated strongly with increased 30‐day mortality (HR: 2.291, 95% CI 1.260‐4.168), 1‐year mortality (HR: 1.933, 95% CI 1.316‐2.840) and 3‐year mortality (HR: 1.743, 95% CI 1.235‐2.461). Kaplan–Meier curves demonstrated that, regardless of SBP or DBP, the long‐term survival probabilities of patients in the highest ARV group were significantly lower than that of those in other groups. RCS curves showed that the death risk of patients with AMI first decreased and then increased with the increase in ARV when DBP‐ARV < 8.04. The 30‐day death risk first increased and then decreased, and the 1‐year and 3‐year death risks increased and then stabilized with ARV increase when DBP‐ARV > 8.04.

**Conclusion:**

This study showed that patients with AMI may have an increased risk of short‐ and long‐term death if their DBP‐ARV is higher or lower during the first 24‐h in ICU.

## Graphical abstract

Key findings:

- A simple and effective indicator of high mortality risk in AMI patients is needed.
- The relationship between BPV and AMI is poorly understood and controversial.
- We show that DBP‐ARV is significantly associated with short‐ and long‐term mortality.
- SBP‐ARV is not significantly associated with mortality risk in patients with AMI.

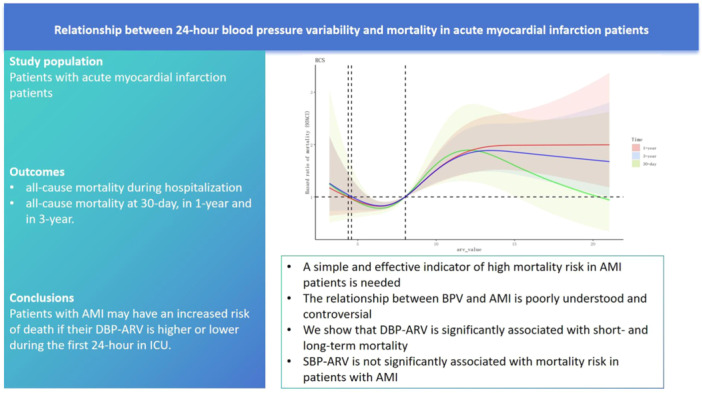

**Abbreviations**
AMI: acute myocardial infarction
ARV: average real variability
BPV: blood pressure variability
CI: confidence interval
DBP: diastolic blood pressure
HR: hazard ratio
ICU: intensive care unit
MACEs: major adverse cardiovascular events
PCI: percutaneous coronary intervention
SBP: systolic blood pressure
SDdn: average SD during the day and night

## 1 INTRODUCTION

Cardiovascular disease accounts for over 40% of all deaths in China, and both the morbidity and mortality related to cardiovascular diseases continue to increase.^1^ The estimated number of patients with coronary heart disease is as high as 11 million.^1^ Acute myocardial infarction (AMI) is a coronary heart disease resulting in a critical condition and poor prognosis. Although percutaneous coronary intervention (PCI) and other revascularization therapies have improved the in‐hospital mortality and long‐term prognosis of AMI, the in‐hospital mortality of patients with AMI has not declined significantly in recent years.^2^ Preventing recurrent heart attacks and other cardiovascular events is crucial for improving patients’ survival and quality of life. Therefore, identifying a simple and effective indicator of high mortality risk in critically ill patients with AMI is imperative to allow early intervention.

Blood pressure variability (BPV) may be related to higher mortality in patients with AMI,^3^ and the relationship between BPV and mortality in patients with AMI is increasingly of interest. BPV, which refers to changes in blood pressure over a certain period, results from interactions between hemodynamics, neurohumoral regulation, behavior, and environmental factors. The relationship between BPV and all‐cause mortality and major adverse cardiovascular events (MACEs) remains controversial, with inconsistent research findings. Some studies have shown that BPV predicts cardiovascular events independently of mean blood pressure,^4^, ^5^ and that it is a better predictor than the mean blood pressure level.^6^ Basson et al.^7^ demonstrated a correlation between long‐term BPV and the incidence of adverse events, such as death and heart failure, independent of hypertension.^8^ However, several studies have indicated no significant association between long‐term BPV and cardiovascular events^9^, ^10^ or an increased risk of cardiovascular events is related to long‐term BPV only in patients with hypertension.^11^ Furthermore, a few studies have shown that short‐term BPV may better predict adverse cardiovascular events in young patients with hypertension, as well as mortality in older patients with hypertension.^12^, ^13^

Most patients with AMI have endothelial dysfunction, which primarily manifests as atherosclerosis. Endothelial dysfunction and atherosclerosis can cause vascular baroreflex dysfunction, leading to blood pressure instability and increased BPV.^14^, ^15^ Abnormal fluctuations in blood pressure can trigger a series of pathophysiological changes, impair blood vessels (including the coronary arteries), increase cardiac load and oxygen demand, affect cardiac structure and function in patients with AMI, damage target organs,^14^, ^15^, ^16^ and subsequently affect prognosis. The impact of BPV on the outcomes of patients with AMI may be a major concern in AMI treatment. However, studies on the relationship between BPV and AMI prognosis have been limited, with contradicting results.

This study investigated the relationship between short‐term BPV and short‐ and long‐term mortality in patients with AMI.

## 2 MATERIALS AND METHODS

### 2.1 Study design

We obtained all the data from the MIMIC‐IV 2.0 database and conducted a retrospective cohort study. This database is maintained by the Beth Israel Deaconess Medical Center and the Massachusetts Institute of Technology and includes the medical information of patients admitted to the intensive care unit (ICU) between 2008 and 2019. We obtained permission to access the data after applying for and completing the Collaborative Institutional Training Initiative course and testing (Record ID: 50038981). Owing to the anonymized nature of all patient information in the database, the institutional review board of the Beth Israel Deaconess Medical Center granted a waiver for obtaining informed patient consent and approved the data‐sharing initiative.

### 2.2 Study population

Patient information was obtained from the MIMIC‐IV 2.0 database. The inclusion criteria were as follows: (1) diagnosis of AMI, (2) age ≥18 years, and (3) first ICU admission. Exclusion criteria were as follows: (1) ICU length of stay <1 day; (2) more than 1 h interval between consecutive blood pressure measurements within the first 24 h of ICU admission, with fewer than 24 blood pressure measurements recorded; (3) systolic blood pressure (SBP) <30 or >300 mmHg, or diastolic blood pressure (DBP) <20 or >200 mmHg.

### 2.3 Data extraction

This study extracted the following patient data from the MIMIC‐IV 2.0 database: age, sex, race, ICU length of stay, comorbidities, clinical conditions (hypertension, diabetes, acute heart failure, cardiogenic shock, cardiac arrest), initial 24 h blood pressure recordings after admission, and survival outcomes (all‐cause mortality at 30 days, 1 year, and 3 years). We used the International Classification of Diseases, 9th and 10th editions (ICD‐9 and ICD‐10) to classify all disease diagnoses. The Navicat software was used to extract data.

### 2.4 BPV

The average real variability (ARV) considers the sequence of measurements and is calculated based on the differences between consecutive blood pressure measurements. In this study, BPV was assessed using ARV, calculated using the following formula^17^:

$$\mathrm{ARV}=\frac{1}{\sum w}\times \sum_{k=1}^{N‐1}w\times |{\mathrm{BP}}_{k+1}-{\mathrm{BP}}_{k}|,$$

where *N* represents the number of valid blood pressure measurements in the data corresponding to a given subject, with *k* ranging from 1 to *N* − 1 and *w* represents the time interval between two consecutive blood pressure recordings.

### 2.5 Outcomes

The primary outcome measure was all‐cause mortality. We defined 30‐day mortality as short‐term mortality, and 1‐ and 3‐year mortality as long‐term mortality.

### 2.6 Statistical analysis

Continuous variables following a normal distribution are expressed as mean and SD ($\bar{x}\pm s$) and were compared using *t* tests. Non‐normally distributed continuous variables are expressed as the median and interquartile range and were compared between groups using the Kruskal–Wallis test. Categorical variables were presented as counts and percentages and were compared using the chi‐square test. The relationship between BPV and mortality was assessed using Cox proportional hazards regression analysis. The results of Cox proportional hazards regression analysis were represented by hazard ratios (HRs) with 95% confidence intervals. All‐cause mortality was the outcome variable, ARV was the independent variable, and demographic characteristics and baseline information were included as covariates. Variables associated with mortality in univariate analysis (*p* < .1) were included as covariates in further multivariate analyses as covariates for further analysis. In the multivariate regression analysis, covariates such as age, hypertension, acute heart failure, cardiogenic shock, cardiac arrest, and PCI were gradually added to the base model to adjust for confounding factors. Sex and diabetes, which are important clinical covariates, were also included in the final model. All covariates and independent variables underwent collinearity tests. Cumulative mortality risk for the four groups was presented using Kaplan–Meier curves. To explore the possible nonlinear relationship between BPV and mortality, we conducted a simulation with restricted cubic spline (RCS) curves and carried out nonlinear tests. Statistical analysis was performed using SPSS (IBM SPSS Inc.) and R statistical software (https://www.r-project.org/) was used for data visualization. A two‐sided *p* < .05 was considered statistically significant.

## 3 RESULTS

### 3.1 Population characteristics

**Figure 1:**
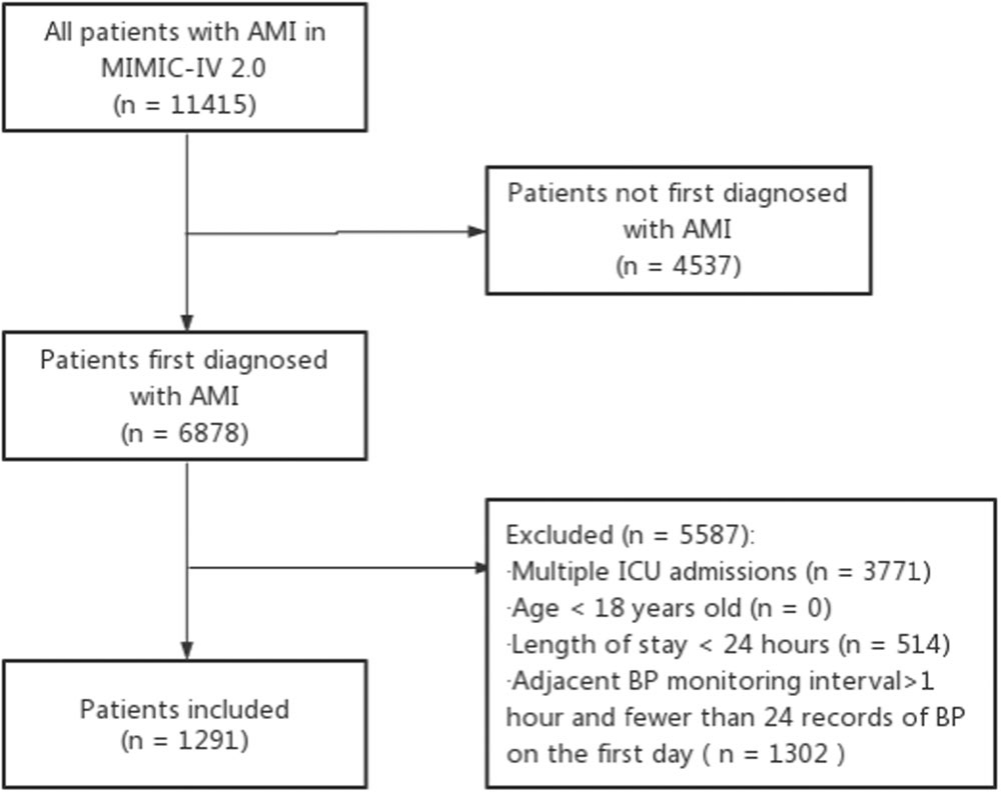
Flowchart for screening patients. AMI, acute myocardial infarction; BP, blood pressure; ICU, intensive care unit.

A total of 11 415 patients with AMI were screened from the MIMIC‐IV 2.0 database and 10 124 patients were subsequently excluded based on the study criteria, resulting in a final study population of 1291 patients (Figure 1). The study population characteristics are summarized in Table 1. The median age of the study population was 71 years and 36.8% of the patients were female. The prevalence of hypertension, diabetes, acute heart failure, cardiogenic shock, and cardiac arrest in the study population was 43.5%, 16.3%, 17.7%, 11.7%, and 4.6%, respectively. Approximately 47.6% of the patients underwent PCI.

**Table 1:** Baseline characteristics.

	Quartiles of ARV of SBP					Quartiles of ARV of DBP				
	Q1 (≤7.77)	Q2 (7.77–9.76)	Q3 (9.76–12.34)	Q4 (≥12.34)	*p*	Q1 (≤6.23)	Q2 (6.23–8.02)	Q3 (8.02–10.46)	Q4 (≥10.46)	*p*
*N*	323	323	323	322		323	323	323	322	
Age, years, Median (IQR)	69.0 (58.0, 79.0)	70.0 (60.0, 79.0)	72.0 (63.0, 81.8)	75.5 (66.0, 84.0)	<.001	70.0 (60.0, 78.0)	70.0 (60.0, 78.0)	72.0 (61.0, 81.5)	75.5 (66.0, 84.0)	<.001
Sex, female, *n* (%)	98 (30.3)	105 (32.5)	134 (41.5)	138 (42.9)	.001	87 (26.9)	100 (31.0)	125 (38.7)	163 (50.6)	<.001
Race, *n* (%)					.094					.795
Asian	3 (0.9)	4 (1.2)	6 (1.9)	11 (3.4)		8 (2.5)	6 (1.9)	3 (0.9)	7 (2.2)	
White	217 (67.2)	193 (59.8)	218 (67.5)	205 (63.7)		212 (65.6)	214 (66.2)	203 (62.8)	204 (63.3)	
Black	17 (5.3)	25 (7.7)	21 (6.5)	27 (8.4)		20 (6.2)	18 (5.6)	27 (8.4)	25 (7.8)	
Other	86 (26.6)	101 (31.3)	78 (24.1)	79 (24.5)		83 (25.7)	85 (26.3)	90 (27.9)	86 (26.7)	
Comorbidities, *n* (%)										
Hypertension	124 (38.4)	141 (43.7)	153 (47.4)	144 (44.7)	.133	133 (41.2)	128 (39.6)	152 (47.1)	149 (46.3)	.148
Diabetes mellitus	47 (14.6)	46 (14.2)	52 (16.1)	65 (20.2)	.150	58 (18.0)	47 (14.6)	48 (14.9)	57 (17.7)	.507
Acute heart failure	69 (21.4)	65 (20.1)	50 (15.5)	44 (13.7)	.030	53 (16.4)	67 (20.7)	52 (16.1)	56 (17.4)	.389
Cardiogenic shock	43 (13.3)	38 (11.8)	30 (9.3)	40 (12.4)	.424	34 (10.5)	43 (13.3)	38 (11.8)	36 (11.2)	.722
Cardiac arrest	11 (3.4)	20 (6.2)	17 (5.3)	11 (3.4)	.232	17 (5.3)	15 (4.6)	16 (5.0)	11 (3.4)	.693
Laboratory parameters										
HGB (g/dL)	12.1 (10.4, 13.6)	12.3 (10.3, 13.8)	12.0 (10.4, 13.4)	11.5 (9.8, 13.3)	.004	12.1 (10.5, 13.5)	12.3 (10.3, 13.7)	12.2 (10.2, 13.7)	11.7 (9.9, 13.3)	.010
Platelet (×10^9^/L)	220.5 (174.0, 265.0)	215.0 (173.0, 268.0)	214.5 (177.0, 271.8)	214.0 (171.0, 268.0)	.830	204.5 (165.5, 257.8)	215.0 (174.0, 267.3)	224.0 (180.5, 277.0)	217.0 (174.3, 270.8)	.041
TNT (ng/mL)	1.45 (0.54, 4.96)	1.17 (0.38, 3.73)	0.86 (0.25, 12.98)	0.64 (0.21, 2.11)	<.001	1.33 (0.43, 3.55)	0.89 (0.29, 3.28)	0.96 (0.27, 3.71)	0.89 (0.30, 2.51)	.093
Cr (mg/dL)	1.0 (0.8, 1.4)	1.0 (0.8, 1.4)	1.1 (0.8, 1.6)	1.1 (0.9, 1.7)	.075	1.1 (0.9, 1.6)	1.0 (0.8, 1.5)	1.0 (0.8, 1.5)	1.1 (0.8, 1.6)	.044
GLU (mg/dL)	132.5 (109.3, 173.3)	133.0 (109.0, 177.0)	133.0 (109.0, 183.8)	136.0 (112.0, 193.0)	.700	136.0 (111.0, 182.8)	132.5 (108.0, 175.0)	133.0 (111.0, 184.0)	135.0 (113.0, 181.8)	.692
PCI, *n* (%)	175 (54.2)	156 (48.3)	142 (44.0)	141 (43.8)	.026	152 (47.1)	144 (44.6)	161 (49.8)	157 (48.8)	.564
Outcomes										
30‐day mortality, *n* (%)	33 (10.2)	28 (8.7)	29 (9.0)	42 (13.0)	.242	25 (7.7)	24 (7.4)	41 (12.7)	42 (13.0)	.020
1‐year mortality, *n* (%)	66 (20.4)	66 (20.4)	72 (22.3)	93 (28.9)	.032	56 (17.3)	57 (17.6)	79 (24.5)	105 (32.6)	<.001
3‐year mortality, *n* (%)	76 (23.5)	87 (26.9)	85 (26.3)	108 (33.5)	.033	71 (22.0)	69 (21.4)	96 (29.7)	120 (37.3)	<.001

Abbreviations: ARV, average real variability; Cr, creatine; DBP, diastolic blood pressure; GLU, glutamine; HGB, hemoglobin; IQR, interquartile range; PCI, percutaneous coronary intervention; Q, quartile; SBP, systolic blood pressure; TNT, troponin T.

Patients were categorized into four groups based on the quartiles of SBP‐ARV and DBP‐ARV within the first 24 h of ICU admission. Overall, baseline characteristics were comparable among the different groups, but significant differences in terms of age, sex, and hemoglobin levels were observed among both the SBP and DBP groups (Table 1). Moreover, significant differences were found in acute heart failure, troponin T levels, and PCI treatment among the SBP groups, and in platelet count and serum creatinine levels among the DBP groups. The group with the highest ARV quartile for both SBP and for DBP included older patients, a higher proportion of females, a lower incidence of acute heart failure, and lower hemoglobin levels (Table 1).

### 3.2 Relationship between BPV and mortality in patients with AMI

Of the included patients with AMI, 132 (10.2%) died within 30 days, 297 (23.0%) died within 1 year (23.0%), and 356 (27.6%) died within 3 years. These findings indicated that the mortality rate in patients with AMI remains high. Comparing the mortality among different groups, we found that patients in the highest SBP‐ARV and DBP‐ARV groups had a significantly higher mortality risk than those in the lowest SBP‐ARV and DBP‐ARV groups.

The results of univariate analysis showed that, compared to the lowest (Q1) group of SBP‐ARV, the highest SBP‐ARV quartile (Q4) had some predictive ability for 1‐ and 3‐year mortality (Table 2). Similarly, compared with the lowest (Q1) group of DBP‐ARV, the higher quartiles (Q3 and Q4) of DBP‐ARV had some predictive ability for 30‐day, 1‐year, and 3‐year mortality (Table 2).

**Table 2:** Multivariable analysis of the association between SBP‐ARV and DBP‐ARV and mortality.

Outcomes	Groups	SBP					DBP				
		Mortality, % (*n*)	Univariable model		Multivariable model		Mortality, % (*n*)	Univariable model		Multivariable model	
			HR (95% CI)	*p*	HR (95% CI)	*p*		HR (95% CI)	*p*	HR (95% CI)	*p*
30‐day mortality	Q1	10.2% (33)	Reference	–	–	–	7.7% (25)	Reference	–	–	–
	Q2	8.7% (28)	0.846 (0.511–1.399)	.514	0.842 (0.461–1.537)	.575	7.4% (24)	0.948 (0.542–1.660)	.852	0.737 (0.368–1.477)	.390
	Q3	9.0% (29)	0.879 (0.534–1.448)	.614	1.030 (0.570–1.862)	.922	12.7% (41)	1.679 (1.021–2.762)	.041	1.898 (1.037–3.474)	.038
	Q4	13.0% (42)	1.314 (0.833–2.073)	.241	1.148 (0.645–2.044)	.638	13.0% (42)	1.738 (1.059–2.852)	.029	2.291 (1.260–4.168)	.007
1‐year mortality	Q1	20.4% (66)	Reference	–	–	–	17.3% (56)	Reference	–	–	–
	Q2	20.4% (66)	0.987 (0.702–1.388)	.939	0.935 (0.627–1.394)	.741	17.6% (57)	1.012 (0.700–1.463)	.951	0.819 (0.528–1.270)	.372
	Q3	22.3% (72)	1.083 (0.776–1.513)	.638	0.998 (0.673–1.480)	.993	24.5% (79)	1.472 (1.045–2.073)	.027	1.424 (0.953–2.128)	.085
	Q4	28.9% (93)	1.481 (1.081–2.031)	.015	1.058 (0.719–1.559)	.774	32.6%(105)	2.036 (1.472–2.816)	<.001	1.933 (1.316–2.840)	.001
3‐year mortality	Q1	23.5% (76)	Reference	–	–	–	22.0% (71)	Reference	–	–	–
	Q2	26.9% (87)	1.136 (0.835–1.545)	.418	1.067 (0.745–1.529)	.722	21.4% (69)	0.965 (0.693–1.343)	.831	0.814 (0.553–1.198)	.297
	Q3	26.3% (85)	1.117 (0.819–1.521)	.485	1.025 (0.714–1.471)	.894	29.7% (96)	1.424 (1.048–1.935)	.024	1.324 (0.925–1.896)	.125
	Q4	33.5% (108)	1.513 (1.128–2.029)	.006	1.098 (0.770–1.564)	.606	37.3% (120)	1.872 (1.395–2.510)	<.001	1.743 (1.235–2.461)	.002

Abbreviations: ARV, average real variability; CI, confidence interval; DBP, diastolic blood pressure; HR, hazard ratio; Q, quartile; SBP, systolic blood pressure.

**Figure 2:**
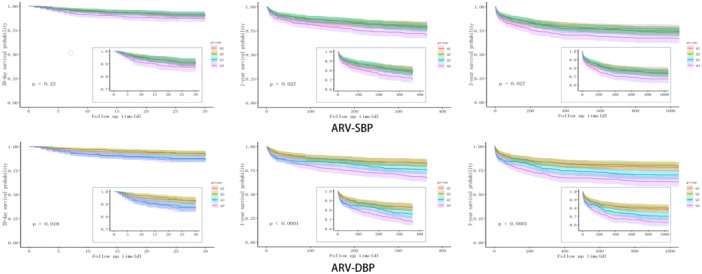
Kaplan–Meier survival curves of patients according to systolic (SBP) and diastolic blood pressure (DBP) average real variability (ARV).

To eliminate the influence of confounding factors on mortality, a multivariable Cox proportional hazards model was used to analyze 30‐day, 1‐year, and 3‐year mortality rates. The results showed that, compared to the lowest (Q1) quartile of ARV, no significant correlation existed between SBP‐ARV and increased risk of death in patients with AMI (Table 2). However, the highest (Q4) quartile of DBP‐ARV still had a higher predictive ability for 30‐day, 1‐year, and 3‐year mortality, with an increased predictive ability for short‐term mortality and a slight decrease in the predictive ability for long‐term mortality (Table 2). For every 1 SD increase in ARV, the risk of death in AMI patients increased by approximately two times. The Kaplan–Meier curves demonstrated that regardless of SBP or DBP, the 1‐ and 3‐year probabilities of survival in the highest (Q4) quartile groups of ARV were significantly lower than those in the other quartile groups (Figure 2).

### 3.3 Nonlinear relationship between DBP‐ARV and AMI mortality

**Figure 3:**
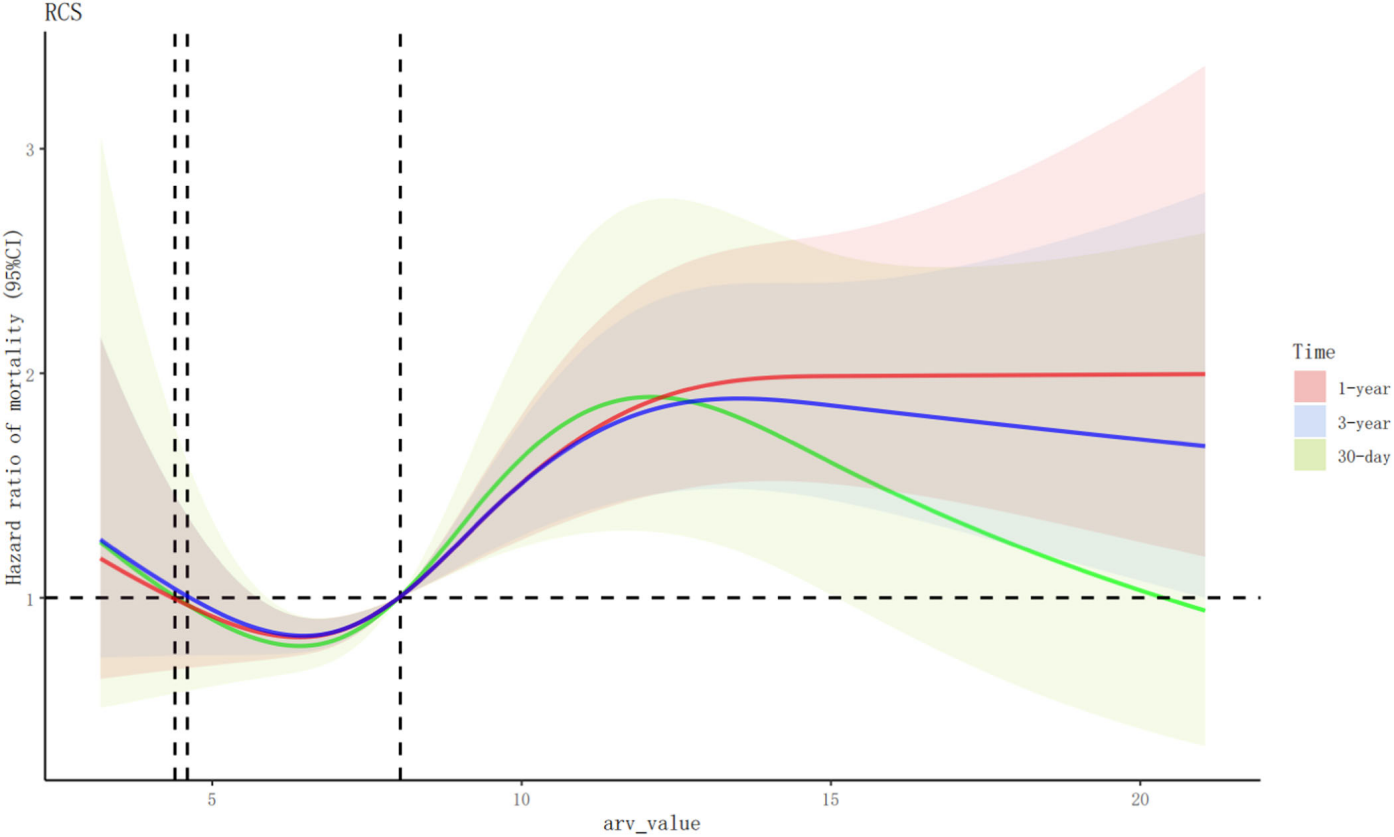
Restricted cubic spline (RCS) curves of mortality risk according to diastolic blood pressure average real variability (ARV) values. CI, confidence interval.

When we compared the mortality among different DBP‐ARV groups, we found that the 30‐day and 3‐year mortality of patients with AMI did not increase with an increase in DBP‐ARV. To explore the possible nonlinear relationship between ARV and mortality further, a simulation was conducted based on RCS curves (Figure 3). We showed that, when the HR= 1, ARV = 8.04. Thus, when DBP‐ARV < 8.04, the short‐term and long‐term death risk of patients with AMI decreased first and then increased with an increase in ARV. When DBP‐ARV = 6.42, the patients’ death risks of 30 days (nonlinear test *p* = .017) and 1 year (nonlinear test *p* = .009) were the lowest. When the DBP‐ARV was 6.51, the 3‐year death risk (nonlinear test, *p* = .002) was the lowest. When the DBP‐ARV was >8.04, the 30‐day death risk of patients with AMI first increased and then decreased with an ARV increase. Additionally, the 1‐ and 3‐year death risks increased and stabilized with an increase in ARV. When the DBP‐ARV was 12.07, the 30‐day mortality risk was the highest. When the DBP‐ARV was 13.51, the 3‐year death risk was the highest.

## 4 DISCUSSION

This study investigated the association between BPV during the first 24 h after admission to the ICU and short‐ and long‐term mortality in 1291 patients with AMI. The results indicated that DBP‐ARV was significantly associated with short‐ and long‐term mortality, demonstrating a nonlinear relationship with mortality, whereas SBP‐ARV was not significantly associated with an increased mortality risk in patients with AMI.

Previous studies on the relationship between BPV and cardiovascular outcomes have primarily focused on hypertensive populations.^18^, ^19^ However, emerging evidence suggests that BPV is independently associated with target organ damage, cardiovascular events, and risk of death in patients without hypertension, including those with diabetes, chronic kidney disease, and obstructive sleep apnea.^20^, ^21^, ^22^, ^23^ In recent years, the relationship between BPV and the prognosis of patients with AMI has garnered attention.

A previous prospective study investigated cardiovascular outcomes during hospitalization in patients with acute coronary syndrome (ACS) by evaluating the average hourly values of the SD of BPV,^24^ which were corrected for day–night differences and were expressed as the average SD during the day and night (SDdn). The SDdn eliminates the influence of diurnal blood pressure variations on BPV.^24^ The results of that study revealed that systolic and diastolic BPV SDdn were the only independent predictors of in‐hospital MACEs in patients with ACS, either with or without ST‐segment elevation myocardial infarction, in those with and without hypertension.^24^ Receiver operating characteristic curve analysis showed that SBP‐SDdn had the highest specificity (98.4%) for predicting MACEs in hospitals, with a sensitivity of 47.3% at a threshold of 12.6 mmHg.^24^ These findings were consistent with the results of the present study, which demonstrated that DBP‐ARV is an important contributing factor to increased mortality risk in patients with AMI. Additionally, a previous retrospective study indicated no significant correlation between 24 h SBP and DBP SD and ARV, and the occurrence of in‐hospital MACEs in patients with AMI.^25^ Similarly, the present study showed no significant correlation between the SBP‐ARV and the risk of short‐ and long‐term death in patients with AMI.

In comparison to short‐term BPV, Choo et al.^3^ suggested that long‐term variation independent of mean blood pressure (VIM) was associated with long‐term outcomes in patients with AMI after PCI. The results indicated that both systolic and diastolic VIM were correlated with adverse cardiovascular events in patients with AMI and improved the predictive ability of the Global Registry of Acute Coronary Events (GRACE) risk score for all‐cause mortality and MACEs. However, diastolic VIM was the only independent predictor of all‐cause mortality and MACEs. In the present study, we found that the highest DBP‐ARV quartile group had higher short‐ and long‐term mortality rates than the lowest DBP‐ARV quartile group. These results indicated that DBP‐ARV is an important predictor of short‐ and long‐term mortality in patients with AMI. Logistic regression analysis revealed that age, hypertension, acute heart failure, cardiogenic shock, cardiac arrest, and PCI status were independent risk factors for mortality in patients with AMI. The Kaplan–Meier curves demonstrated a significantly higher survival probability in the lowest than in the highest DBP‐ARV group.

Currently, no consensus regarding the appropriate BPV range has been reached. Exploring the relationship between BPV and prognosis in patients with AMI is important for improving patient outcomes. Some researchers have investigated the association between mean arterial pressure ARV and the risk of all‐cause mortality in critically ill patients without AMI. The relationship is U‐shaped. They found that patients with higher mean arterial pressure ARV had an increased risk of all‐cause mortality when ARV ≥ 7.2 mmHg, whereas in those with ARV < 7.2 mmHg, a lower BPV may be a risk factor.^17^ In the present study, we identified a nonlinear relationship between DBP‐ARV and mortality in patients with AMI using RCS curves. When DBP‐ARV was <8.04, the short‐ and long‐term mortality risks first decreased and then increased with increasing ARV. When DBP‐ARV was >8.04, the 1‐ and 3‐year mortality risks increased with higher ARV levels, reaching a plateau.

However, the pathogenesis and mechanisms of BPV infection remain unclear and require further investigation. Studies have shown that BPV is associated with arterial remodeling, arteriosclerosis, vascular damage, and endothelial dysfunction.^26^, ^27^, ^28^, ^29^, ^30^ Furthermore, BPV, arterial remodeling, and arteriosclerosis mutually influence each other, as increased BPV can promote arterial remodeling and arteriosclerosis, whereas arterial remodeling and arteriosclerosis can also increase BPV.^31^ Animal experiments have shown that BPV can stimulate cardiac vascular smooth muscle cell proliferation, extracellular matrix deposition, fibrosis, and microvascular changes, thereby affecting cardiac vasculature structure and function.^32^ Clinical studies have demonstrated an association between BPV in early adulthood and cardiac structure and function in later life.^33^ This suggests that BPV changes may occur before and could be used to predict the risk of left ventricular remodeling in patients. These studies indicate that elevated BPV can alter vascular and cardiac structures, leading to cardiovascular diseases.

Although the present study explored the correlation between the 24 h ARV and short‐term and long‐term mortality in patients with AMI, certain limitations remain. First, this was a retrospective study with an inevitable selection bias. Second, due to insufficient or undisclosed information in the database, potential confounding factors, such as echocardiographic findings, GRACE score, and Killip classification were not included. Additionally, different time intervals may exist between pairs of blood pressure records in the database. Lastly, the present study only investigated the relationship between BPV during the first 24 h in the ICU and mortality without considering the potential relationship between long‐term BPV and mortality.

## 5 CONCLUSION

We found a significant correlation between the first 24 h diastolic BPV in patients with AMI admitted to the ICU and increased short‐ and long‐term mortality risk. When DBP‐ARV < 8.04, the risk of mortality in patients with AMI decreased first and then increased with an ARV increase. On the other hand, when DBP‐ARV > 8.04, the 30‐day mortality risk first increased and then decreased with an ARV increase, whereas the 1‐ and 3‐year mortality risks increased with an increase in ARV and then stabilized after reaching a certain level. Thus, this study provided insights into the relationship between BPV and mortality risks in patients with AMI. Further clinical research is needed to explore the relationship between long‐term BPV and prognosis in patients with AMI, to compare the predictive value of short‐ and long‐term BPV for cardiovascular events and mortality risk, and to elucidate the underlying mechanisms of BPV.

## AUTHOR CONTRIBUTIONS

**Ying Liu**: Research design, data extraction, data analysis, and paper writing. **Wen Li, Xinju Liu, Mengxue Hei**: Data extraction. **Shuoyan An, Zhengqin Zhai**: Validation and editing; **Gailing Chen**: Study design, administrative support, and manuscript revision. All authors have read and agreed to publish the final manuscript.

## CONFLICT OF INTEREST STATEMENT

The authors declare no conflict of interest.

## Data Availability

Publicly available data sets were analyzed in this study. These data are here at https://physionet.org/content/mimiciv/2.0/.
